# A Comparison of Two Sensory Panels Trained with Different Feedback Calibration Range Specifications via Sensory Description of Five Beers

**DOI:** 10.3390/foods8110534

**Published:** 2019-11-01

**Authors:** Line Elgaard, Line A. Mielby, Helene Hopfer, Derek V. Byrne

**Affiliations:** 1 Department of Food Science, Faculty of Science and Technology, Aarhus University, Kirstinebjergvej 10, DK-5792 Aarslev, Denmark; lineh.mielby@food.au.dk (L.A.M.); derekv.byrne@food.au.dk (D.V.B.); 2 Department of Food Science and Sensory Evaluation Center, The Pennsylvania State University, University Park, State College, PA 16802, USA; hxh83@psu.edu

**Keywords:** sensory descriptive analysis, panel performance, training, Feedback Calibration Method, beer

## Abstract

Feedback on panel performance is traditionally provided by the panel leader, following an evaluation session. However, a novel method for providing immediate feedback to panelists was proposed, the Feedback Calibration Method (FCM). The aim of the current study was to compare the performance of two panels trained by using FCM with two different approaches for ranges calibration, namely self-calibrated and fixed ranges. Both panels were trained using FCM for nine one-hour sessions, followed by a sensory evaluation of five beer samples (in replicates). Results showed no difference in sample positioning in the sensory space by the two panels. Furthermore, the panels’ discriminability was also similar, while the self-calibrated panel had the highest repeatability. The results from the average distance from target and standard deviations showed that the self-calibrated panel had the lowest distance from target and standard deviation throughout all sessions. However, the decrease in average distance from target and standard deviations over training sessions was similar among panels, meaning that the increase in performance was similar. The fact that both panels had a similar increase in performance and yielded similar sensory profiles indicates that the choice of target value calibration method is unimportant. However, the use of self-calibrated ranges could introduce an issue with the progression of the target scores over session, which is why the fixed target ranges should be applied, if available.

## 1. Introduction

Sensory descriptive analysis (DA) is an essential and crucial tool in the field of sensory science [1,2]. Since its invention in the 1940s, descriptive analysis has evolved into several different types of sensory descriptive profiling methods [3,4]. Besides generic descriptive analysis [1,2], there are several specific versions, such as the Flavor Profile [5], the Texture Profile [6], the Spectrum™ method [2], and Quantitative Descriptive Analysis (QDA) [7,8]. Common to all methods are vocabulary generation and training of the DA panel. 

Regardless of the variation, a DA panel requires an extensive amount of training to become a reliable sensory instrument. Up to 130 h of training and more are reported [6], depending on the chosen method, the complexity of the product, the number of attributes, and the test sensitivity requirements [1,2,3,4]. Studies have shown that different factors related to the training step can influence the performance of a sensory panel [9,10,11,12,13,14,15]; these include, among others, the panel’s sensory experience, product knowledge, and product involvement. During DA training, a common vocabulary and reference frame are established, together with procedures for evaluation and training in the use of the scale. Various vocabularies and reference frames were developed for a broad range of food products [16,17,18,19,20,21,22,23], to facilitate communication about sensory properties. However, often these need to be adapted and further refined for the specific product to be evaluated. 

Once vocabularies and reference frames are established, DA training continues to ensure that all the panelists understand and consistently and reliably use these common concepts. During training, the panel leader provides the panelists with individual feedback in relation to the overall panel performance, to reduce individual differences between panelists. Types of performance feedback often include, but are not limited to, measurements of repeatability, discriminability, scale usage, and conceptual understanding of attributes. Similarly, analysis of panel group performance and performance monitoring is communicated to panelists in various ways, either with numbers and statistical results or graphically and with focus on consensus, repeatability, and discriminability [24,25,26,27]. The main goal of this feedback is to improve product evaluation performance; it is not necessarily optimized to provide the most efficient feedback that would allow panelists to readily incorporate that feedback. 

In psychology, the improvement of sensory abilities is termed perceptual learning. Perceptual learning is defined as the process of long-term improvement of one’s perceptual abilities by practice in perceptual tasks [28]. 

Prior studies, most of them focusing on visual learning, have investigated the effect of different types of feedback, as well as timing of feedback, on both learning in general and perceptual learning specifically [29,30,31,32,33,34]. Herzog and Fahle [30] found that feedback in the form of trial-by-trial (i.e., feedback following each assessment) and block feedback (i.e., percentage of correct responses) significantly increased the overall number of correct responses over the experimental replications. Meanwhile, the conditions with incorrect or no feedback at all generally showed no overall significant improvement in performance, with participants having either increasing, oscillating/fluctuating, or decreasing performances. However, the authors argue that learning without feedback might be possible, though at a slower pace than with correct feedback (trial-by-trial or block). 

Similarly, Bangert-Drowns et al. [29] studied the effectiveness of feedback through a meta-analysis of previous studies and found that eight variables significantly influenced the effectiveness of feedback, including type of feedback (right–wrong/correct answer/repeat until correct/explanation) and timing of feedback (immediate after item/immediate after test/delayed after test). Information about right/wrong was less effective compared to information about the correct answer. Furthermore, immediate feedback was more effective than delayed feedback. Similarly, Kulik and Kulik [31] also explored the effect of immediate versus delayed feedback and concluded that the type of feedback is related to the nature of the task. Delayed feedback only increased learning in specific experimental situations (e.g., list-learning), while immediate feedback was most effective in almost all other experimental situations. Trowbridge and Cason [33] investigated the effect of four different types of feedback on improvement for a line-drawing task (drawing a fixed line length). The results showed that informing the participants about the distance of their line from the correct line length was the most effective type of feedback, followed by information about right–wrong length. The lowest effectiveness was found with no feedback provided and when the experimenter spoke nonsense words as a distraction. 

Altogether, this body of research seems to suggest that providing participants with feedback is more effective than providing no feedback at all. Furthermore, the effectiveness of feedback seems to be related to both the timing and type of feedback, with immediate feedback being more effective than delayed feedback, and a tendency for a more descriptive and precise type of feedback being more effective, as well. However, despite the extensive amounts of studies that have investigated general and perceptual learning, very little literature exists regarding perceptual learning in relation to the sense of taste and flavor [35]. 

In a case study on wine, Walk [34] investigated the influence of feedback type on perceptual learning during a same–difference test of wines. They found an overall significant increase in percentage of correct responses from pretest to posttest (with training sessions in-between), independent of the feedback type (information about right–wrong answer, information about right–wrong answer together with information about which wines were correctly/incorrectly identified, and no feedback at all), indicating learning by sheer exposure. The author argues that the lack of difference in results between feedback methods could be related to the length of the experiment and the nature of the task. This is because they train for a total of 20 sessions, which, in many cases, is more than typically used in DA studies [36]. 

Traditionally, participants in a sensory descriptive panel receive feedback about their performance from the panel leader, following the evaluation session. However, since studies outside the field of sensory science have shown that timing of feedback plays an important role in the effectiveness [29,31,32,37,38], a novel approach of providing immediate feedback to panelists was introduced [39,40]. The so-called Feedback Calibration Method (FCM) minimizes the time between sample evaluation and feedback, thereby increasing the effectiveness of the feedback. FCM distinguishes between target scores and target ranges. The target score for each attribute and sample is the exact point on the scale were the sample should be scored. The target range is defined as the allowed range around the target score for each attribute and sample that is considered a correct score, similar to confidence intervals. Feedback is provided to panelists onscreen, immediately following the evaluation of a sample, as an indication of whether the panelist hit or missed the optimal scoring range for each product and attribute. Two different methods for defining target scores exist. The target scores can either be fixed throughout the training sessions or the panel can self-calibrate themselves by changing the target score according to the panel mean from the previous training session. Fixed target scores are used if the optimal target for each attribute and product is known, while self-calibrated target scores are used in cases where the attribute ratings for the products are unknown to both the panel and panel leader.

FCM has been used during training and calibration of sensory panels for a wide range of products [41,42,43,44,45,46,47,48,49,50]. Although all of these studies successfully used FCM, none of them discussed the use of FCM in terms of procedure or compared the use of FCM to the traditional training procedure. Furthermore, only one of these studies reports how they derived their target scores and ranges used in FCM. 

Our previous paper [9] and papers by the developers of the method have investigated the implications of using FCM. Findlay et al. [39] investigated the suitability of FCM for sensory analysis training and found it a useful and effective training method. These results were furthermore supported by Findlay et al. [40], who compared a panel receiving traditional feedback (i.e., at the end of the training session), with a panel using FCM. They concluded that both panels showed similar levels of proficiency and improvement (no difference in training time) and that FCM therefore is a useful tool to improve panel performance in descriptive analysis. Elgaard et al. [9] also found that FCM improved panel performance and concluded that FCM was most efficient for a newly trained panel compared to an extensively trained panel.

**Figure 1:**
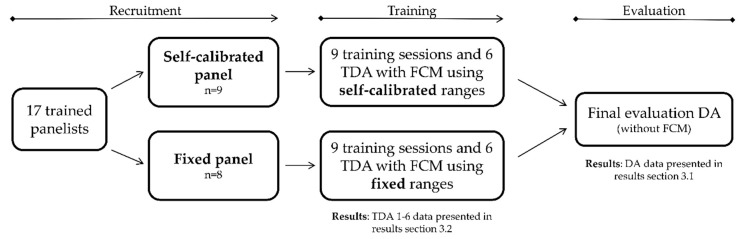
Experimental overview. TDA = training descriptive analysis.

To our knowledge, no studies have investigated how the method for determining target scores and ranges influences panel performance and product evaluation outcome. The nature of the present study provides the researchers with a unique opportunity to investigate this research question. The aim of this study was therefore to investigate the influence of training with FCM, using these two different types of methods for target scores and ranges calibration, namely self-calibrated versus fixed scores and ranges. To answer the aims of this present study, two panels were trained, using fixed and self-calibrated scoring ranges, respectively. Their performance progression during the training sessions and their final sensory evaluation performance were compared. An experimental overview is provided in Figure 1, displaying the steps of recruitment, training, and evaluation.

Four research aims are investigated in this study. The first and second aim are related to the results during the final descriptive analysis, while the third and fourth are related to the performance progression during the training sessions. The first research aim was to investigate if the two different methods (fixed versus self-calibrated) for target score calculation lead to similar or different evaluations of the products in the sensory space. This was investigated by both a vision-based analysis (Generalized Procrustes Analysis) and by numerical values (RV coefficients). The second research aim was to investigate if the two methods yield differences in discriminability and repeatability by investigating F-values and MSE-values, respectively. The third research aim was related to performance during the training sessions and compared the panels’ accuracy through distance from target measurements and precision by standard deviations. The fourth and last aim was to investigate how the mean values for the two panels progress over training sessions, compared to the optimal target value. This was investigated both on an overall-attribute level and on an individual-attribute level, including confidence intervals. The distance from target value is a measure of the panels’ accuracy, while the confidence intervals are a measure of the panels’ precision.

## 2. Materials and Methods 

### 2.1. Samples

Nine pilsner-type beers were included in the present study (see Table 1a for product name, sample code, beer type, and ethanol content (ABV)). All samples were used in the training sessions, but only five of the samples were used in the final evaluation DA sessions (Tuborg, Carlsberg, Jacobsen, Tuborg SL, and Heineken), as they were shown to exhibit diverse sensory profiles [9]. The same five samples were also included for the six training descriptive analysis sessions (TDA 1–6), while the remaining four samples were included in the training sessions to highlight and represent specific attributes. All beers are commercially available and were purchased in DK, Canada, and the United States. To account for batch-to-batch variation, only beers with the same production day/expiry date were used. All samples were stored at 5 °C until immediately before serving.

For product evaluation, beer samples were served monadically in a William Latin Square design within each replication session [51]. To mask the color differences between the beer samples, red light was applied in the booths during testing. The samples (30 mL) were served in 4 oz (~118 mL) clear disposable souffle with lids (Dart, Mason, MI, USA) and coded with 3-digit numbers. The panels had unsalted crackers (Premium, Nabisco, East Hanover, NJ, USA) and DI water as palate cleaners. The samples were all poured simultaneously to account for loss of carbonation and temperature. The serving temperature ranged from 8 to 13 °C between the first and last served sample. The panels were served a Tuborg sample for calibration before each descriptive analysis; however, this calibration sample was not included for evaluation.

### 2.2. Descriptive Analysis Panels

This study was deemed exempt from IRB overview under the wholesome-foods category (The Pennsylvania State University, protocol # STUDY00008551). A total of 17 panelists (7 males, aged 26–64, mean age 44), all with previous experience with descriptive analysis (1+ study), were randomly divided into two panels. The two panels were trained using two different types of range calibration in the FCM [39,40]. The panel referred to as the fixed panel (*n* = 8), was trained using fixed target ranges, established by an expert beer panel, while the other panel, referred to as the self-calibrated panel (*n* = 9), was trained using self-calibrated target ranges (see also Section 2.3.1).

### 2.3. Training Sessions

Both panels were trained for nine one-hour sessions (T1–T9). For both panels, ballot training was used with predefined attributes and references. The attributes, references, and target scores were established in our previous studies [9]. To assist panelists in establishing a framework for each attribute, reference beers were used during training to establish minimum and maximum intensities for each attribute (Table 1b). An overview of training tasks and FCM training sessions is provided in Table 2. Both panels were trained on the same day, with the fixed panel being trained in the morning and the self-calibrated panel trained in the afternoon, for all training sessions.

#### 2.3.1. Application of FCM

Target scores and ranges for the fixed panel: The target scores for the fixed panel were defined based on results from prior descriptive analysis tests [9]. The target ranges for the fixed panel were calculated as the 90% confidence intervals around the target scores, similar to Findlay et al. [39].

Target scores and ranges for the self-calibrated panel: The first target scores for the self-calibrated panel were calculated based on the results from TDA 1, with the mean values for each attribute being set as the first target scores. The target scores changed after each TDA, according to the change in mean value. The ranges for the self-calibrated panel were defined by the mean value +/– 10 on the scale (0–100), which was equivalent to the size of the ranges for the fixed panel. The usual practice of using the confidence interval was not possible, as the standard deviations were too large and covered almost the whole scale.

FCM was not applied for TDA 1, as the self-calibrated panel needed to generate target scores and ranges in TDA 1. Furthermore, since none of the panels were familiar with the use of FCM, FCM was applied for all attributes, but only for a subset of samples (see Table 2b). The subsets of samples were changing between training session to train the panel in the largest possible span in sensory variation. The choice to select a subset of samples to use with FCM was based on Findlay et al. [40], where the number of times feedback was provided increased over sessions, in order to allow the panelists to become familiar with the method.

### 2.4. Final Evaluation DA

After the nine training sessions, both panels evaluated the five beer samples (Carlsberg, Tuborg, Heineken, Jacobsen, and Tuborg SL), following the same sensory profiling procedure. The samples were evaluated using a 100-point line scale anchored at the scale ends with “a little” to “a lot” (Academic Consortium Compusense Cloud, Compusense Inc., Guelph, Canada). The samples were evaluated in triplicates over three consecutive evaluation days. FCM was not applied during the final evaluation DA.

### 2.5. Data Analysis Method

For the statistical analyses, RStudio (Version 3.4.2, RStudio Inc., Boston, MA, USA), PanelCheck software (Version 1.4.2, Nofima Mat, Ås, Norway), and Compusense (Academic Consortium, Compusense Cloud, Compusense Inc., Guelph, Canada) were used.

#### 2.5.1. Comparison of the Panels’ Positioning of the Samples in the Sensory Space

Generalized Procrustes Analysis (GPA) [52] was performed on the final evaluation DA data to visually compare the panels’ positioning of the five beers in the sensory space. For the GPA, data was averaged over both replicates and panelists, and analyzed with the GPA function in the FactoMineR package [53]. The tolerance level for solution convergence was set to 10^−10^, and the maximum number of iterations was set to 200 and data was scaled to unit variance. A permutation test [54,55] was performed on the GPA solution to investigate the strength of the results with the GPA.test function from the RVAideMemoire package [56]. The number of permutations was set to 500. Last, RV coefficients were extracted from the GPA solution, to numerically investigate the panels’ similarities/differences, as reported by the FactoMineR package [53].

#### 2.5.2. Comparison of the Panels’ Discriminability and Repeatability

To further investigate the panels’ performance during the final evaluation DA, both mean square errors (MSEs) and F-values were extracted from the PanelCheck software (Version 1.4.2, Nofima Mat, Ås, Norway). The F-value is a measure of the panels’ discriminability of the samples, with higher F-values indicating better panel discriminability for a particular attribute. The MSE-value is a measure of panels’ repeatability; thus, a low value indicates a high panel repeatability [27]. To account for not normally distributed data, a generalized linear model (GLM) was fitted with the family type set to quasi-Poisson, and ANOVA analysis were conducted.

#### 2.5.3. Comparison of the Panels’ Attribute Understanding and Use of Scale

The results from the different FCM training sessions was analyzed as distance from target measurements, i.e., accuracy [57], and standard deviations, i.e., precision. This approach was applied to study panel performance (attribute understanding and use of scale) during the training sessions (TDA 2–6). The first training session, TDA 1, did not include FCM, as product mean values were needed for use as target score for the self-calibrated panel, before FCM could be applied. Therefore, the analysis does not include results from TDA 1 for both panels. The distance from target measurements were calculated as the absolute value of the distance between the target value and the measured value. When there were two replicates, the distance from the target was calculated for both replicates, and the average distance value was used. Both the distance from target measurements and standard deviations were averaged over product and panelist, and also for simplicity over attribute, when appropriate. To test the significance of the results, ANOVA and post hoc analysis with Tukey’s method were performed, using the emmeans function from the emmeans package [58]. The results from the pairwise comparisons were displayed by the use of the CLD function [59], which is also from the emmeans package [58].

#### 2.5.4. Comparison of the Panels’ Performance Progression over Training Sessions

The panels’ product mean values (averaged over replicate and panelist) were compared to the optimal target values (fixed targets) over TDA sessions, i.e., accuracy. This was done to investigate how the panels’ scoring of samples for each attribute was progressing in relation to the optimal target values. TDA 1 was again not included, based on the same argument as for the above section. Two products (Jacobsen and Carlsberg) were chosen as examples, as these beers showed diverse sensory profiles. Two different types of plots were constructed, displaying the progression on (1) the overall attribute level and (2) the single attribute level, including confidence intervals, i.e., precision. For plot-type one, line charts with data points were constructed in Excel. For plot-type two, the panel mean values and confidence intervals were first calculated with the groupwiseMean function from the rcompanion package [60] and thereafter plotted with the qplot function from the ggplot2 package [61], including error bars based on the confidence intervals. Four plots were chosen as examples to display the different trends observed in the study.

## 3. Results

For simplicity, the results section is divided into two parts (Figure 1), as the study includes two different datasets. First, the results from the final evaluation DA after the training sessions are presented (Section 3.1). Second, the results from the training descriptive analysis (TDA) sessions during the training are presented (Section 3.2), to answer the remaining research questions and to compare the effect of fixed versus self-calibrated target scores.

### 3.1. Comparing the Results of the Descriptive Analysis Evaluation for the Fixed and Self-calibrated Panel 

#### 3.1.1. Comparison of the Panels’ Positioning of the Products in the Sensory Space

**Figure 2:**
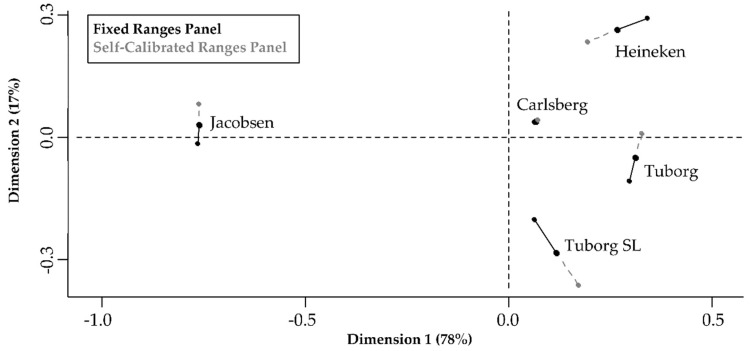
Generalised Procrustes Analysis plot for the positioning of the beers in the sensory space by the two panels.

Generalised Procrustes Analysis (GPA) (Figure 2) was performed to visually investigate the positioning of the five beers in the sensory space by the two panels. The permutation test on the GPA solution resulted in a significantly above-chance result (*p* < 0.01), indicating that the beers were not positioned randomly in the sensory space. The GPA plot shows that all five beer samples were well discriminated from each other by both panels, indicating that both panels could distinguish between the beers. The two panels evaluated the beer samples in a similar manner, since their individual panel means are placed closely together in the sensory space. The Carlsberg beer sample was assessed nearly identically by both panels, while the Tuborg SL beer showed the least agreement between the panels. Nevertheless, the RV coefficient of 0.92 (calculated from the GPA) confirmed the similarity of the two panels, with regard to the sensory profiles of the beers, by being close to 1.

#### 3.1.2. Comparison of the Panels’ Discriminability and Repeatability

**Figure 3:**
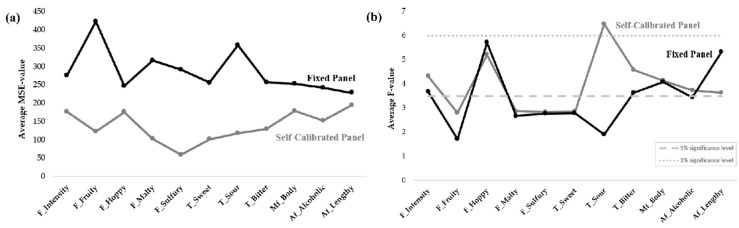
Average (**a**) MSE-values and (**b**) F-values for the two panels. Both plots are averaged over replicate and panelist (F = flavor, T = basic taste, Mf = mouthfeel, Af = aftertaste). The 1% and 5% significance levels are indicated as dotted and dashed lines, respectively.

F-values and MSE-values (Figure 3) were used to assess the panels’ discriminability and repeatability, respectively. For all attributes, except hoppy flavor and lengthy aftertaste, the MSE-values (Figure 3a) were significantly (*p* < 0.05) lower for the self-calibrated panel, compared to the fixed panel. This indicates that the self-calibrated panel was better at replicating itself, compared to the fixed panel. Figure 3a also shows that repeatability was attribute-dependent for both panels, as some attributes appeared to be more difficult to assess than others (e.g., fruity flavor vs. hoppy flavor for the fixed panel, and hoppy flavor vs. sulfury flavor for the self-calibrated panel) and, furthermore, that the panels differed in regard to which attributes they had difficulties in repeating themselves in. The fixed panel showed the highest MSE-values for fruity flavor and sour taste, while the self-calibrated panel had the highest MSE-values for flavor intensity, hoppy flavor, and both the mouthfeel and aftertaste attributes. 

The two panels had similar F-values (Figure 3b) for most attributes, except for sour taste and lengthy aftertaste. The self-calibrated panel had the highest F-value for sour taste, while the fixed panel had the highest for lengthy aftertaste. Despite differences, there was no overall significant effect of panel on F-value (*p*-value for sour was *p* = 0.052). The similar F-values indicate that the panels had a similar discriminability between samples, indicating similar performance. Figure 3b confirmed the attribute dependency found for the MSE-values. For example, both panels showed low discriminability for fruity flavor, but were very discriminating for the hoppy-flavor attribute.

### 3.2. Comparing the Results from Training Sessions, TDA 2 to 6, for the Fixed and Self-calibrated Panel 

#### 3.2.1. Comparison of the Panels’ Distance from Target Measurements

**Figure 4:**
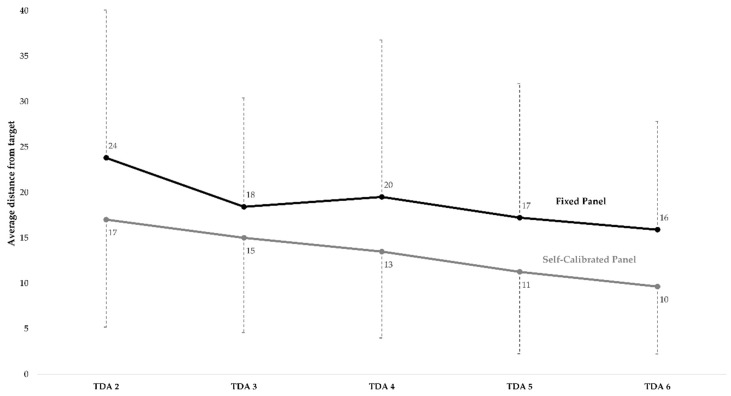
Average distance from target (solid lines) and standard deviations (dashed lines). Data is averaged over product, attribute, and panelist. TDA = training descriptive analysis.

The distance from target measurements and standard deviations were investigated for both panels (Figure 4). Overall, the self-calibrated panel showed the lowest average distance from the target (*p* < 0.001), indicating highest accuracy and overall performance. The self-calibrated panel also had the overall lowest standard deviation, signifying the highest precision. Both panels had a significant (*p* < 0.001) decrease in distance from the target (i.e., significant increase in accuracy) from session TDA 2 to session TDA 6, indicating that both panels learned and improved due to training. The fixed panel had a decrease in average distance from the target (i.e., absolute distance on a 100-point line scale) of 8, while the self-calibrated panel had a decrease of 7. The decrease in distance from target over sessions was similar among the panels, showing a similar performance enhancement regarding accuracy. Both panels also showed a decrease in standard deviation (i.e., increase in precision) over the training sessions.

The distance from the target measurements were also investigated on an individual-attribute level (Table 3). It is shown that differences exist regarding which attributes the two panels showed the largest increase or decrease in performance (i.e., decrease or increase in distance, respectively). A significant effect was found for the interaction between attribute and session (*p* < 0.01), indicating that the distance from the target decreased faster for some attributes compared to others, i.e., an attribute-dependent learning effect was present. The two other interactions (panel/attribute and session/panel) were found to be nonsignificant (*p* > 0.05).

#### 3.2.2. Comparison of the Panels’ Optimal Target Scores and Panel Means Progression Over Training Sessions

**Figure 5:**
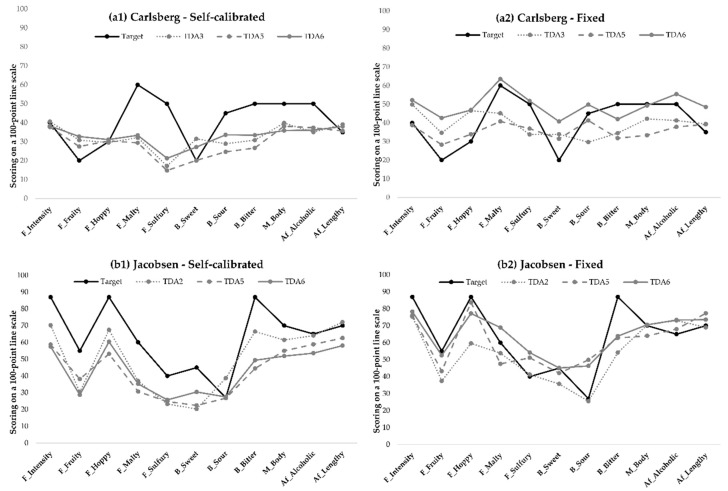
Performance progression (mean scores) compared to the target value over training sessions TDA 2–6 for two products, (**a**) Carlsberg and (**b**) Jacobsen, and both panels, (**b1**) self-calibrated and (**b2**) fixed. Data are averaged over panelist and replicate.

The plots (Figure 5) of the two panels’ mean scores for all attributes and two of the products indicate how the panels’ mean values were progressing over TDA sessions, according to the “optimal scoring targets”. Figure 5a1 shows the self-calibrated panel’s mean scores for all attributes for the Carlsberg product. With increasing training sessions (TDA 3 to TDA 6), the mean score line decreased in slope, indicating that either the self-calibrated panel participants differentiated less between the attributes or they, as a panel, converged around their final target values. The self-calibrated panel hit the target score for the attributes flavor intensity, hoppy flavor, and lengthy aftertaste, and were close to the target for sweet and sour taste. The mean score for fruity flavor moved away from the target score during the training sessions. Furthermore, the mean scores for malty and sulfury flavor, bitter taste, body, and alcoholic aftertaste did not progress toward the target score. In general, most of the mean scores for these attributes did not change much over training sessions.

For the fixed panel in Figure 5a2, there was a clear separation between the three TDA sessions for the Carlsberg sample. The panel hit the target for the attributes malty and sulfury flavor, and body. Additionally, the mean score for sour and bitter taste and alcoholic aftertaste were close to the target. For all of the attributes just mentioned, it was clear that the training sessions helped the panel to progress toward the target. Conversely, for the remaining attributes (five out of 11 total) the mean scores for TDA 6 (solid gray line) moved away from the target, compared to the scores for TDA 2 and TDA 5. This indicates that more training is still needed. 

The plots for the mean scores for the Jacobsen beer sample (Figure 5b1, b2) show that the self-calibrated panel correctly differentiated between, and used, the flavor attributes, but their scores of these attributes were lower than the target score. The self-calibrated panel only hit the target for sour taste, while the fixed panel correctly scored the intensities of fruity flavor, sweet taste, body, and lengthy aftertaste. Additionally, the scores for flavor intensity, hoppy and malty flavor, and alcoholic aftertaste were also close to the target for the fixed panel. The self-calibrated panel only had one attribute for which the panel was moving toward the target (sweet tastes), while the rest of the attributes either stagnated or moved away from the target value as training progressed. The fixed panel’s mean scores were moving toward the target for all attributes over the training sessions, except for sulfury flavor and sour taste. 

**Figure 6:**
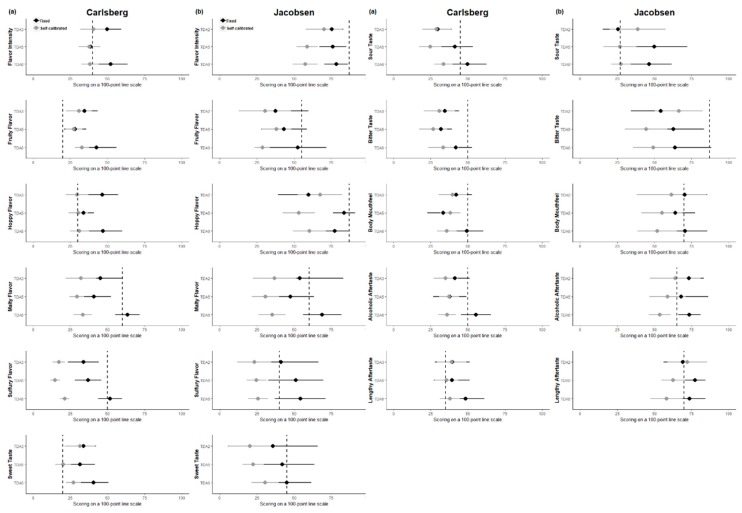
Performance progression (mean scores and confidence intervals) compared to the target value (dashed line), over training sessions TDA 2–6 for (**a**) the Carlsberg sample and (**b**) the Jacobsen sample. Data are averaged over panelists and replicates.

The results regarding mean value progression compared the “optimal target score” were also plotted individually per attribute (Figure 6). Overall, the panels’ progression on an individual-attribute level displayed changes in performance (i.e., accuracy and precision) dependent on attribute, as the effect of training was different for the different attributes over training sessions, i.e., difference in progression behavior. Four plots were chosen to be described in detail as representatives of these differences in behavior, i.e., Carlsberg hoppy flavor, Carlsberg malty flavor, Jacobsen flavor intensity, and Jacobsen sweet taste.

As an example of this behavior, the intensity for hoppy flavor in the Carlsberg sample shows that the self-calibrated panel scored close to the target for all training session (TDA 3–6), indicating a high accuracy, while the fixed panel had trouble scoring this attribute correctly (low accuracy). The self-calibrated panel also showed a decrease in standard deviation over training sessions, indicating an increase in precision. This was, however, not the case for the fixed panel, where the standard deviation grew larger for TDA 6.

For the all of the remaining attribute examples (flavor intensity of the Jacobsen sample, sweet taste of the Jacobsen sample, and malty flavor of the Carlsberg sample), the fixed panel scored closest to the target (highest accuracy), and both panels increased in precision (decrease in standard deviation). For flavor intensity of Jacobsen, the self-calibrated panel again is moved away from the target with increased training, while the fixed panel is moved toward the target. For the intensity of malty flavor attribute for Carlsberg, the self-calibrated panel converged in their mean scores to around 30–35, while the fixed panel moved closer to the target score of 60 during the last training session. Last, for sweet taste of Jacobsen, both panels improved similarly in accuracy and moved closer to the target score over their training session, with the fixed panel hitting the target for the last training session.

## 4. Discussion

### 4.1. The Panels’ Positioning of the Products in the Sensory Space, Their Discriminability, and Repeatability 

Based on the results from the GPA plot, one could argue that the choice of ranges calibration method is less important, as both panels showed similar product differentiation. However, smaller differences between the panels’ evaluations of samples exist. The beer sample with the largest sensory difference between the two panels was Tuborg SL, which showed highest scores for sour taste. For sour taste, the fixed panel displayed a higher MSE-value and lower F-value, indicating that this beer sample was particularly difficult for the fixed panel to assess. 

### 4.2. The Panels’ Attribute Understanding and Use of Scale During Training

The results showed that the self-calibrated panel performed best, both in terms of accuracy and precision (Figure 4). The difference in performance could be related to the nature of the target score definition relative to the size of the target range. This is because the self-calibrated panel participants created their own target scores, while those of the fixed panel were trained on pre-established target scores. The fact that the target scores for the self-calibrated panel were based on panel means could make it easier for the panelists to hit the score, as most of them were already close to the panel mean. In contrast, the fixed panel had to hit pre-established target scores, which were not necessarily close to the panel means. This could potentially have made the target scores more difficult to hit, and thereby increased the fixed panel’s average distance from the target. Furthermore, there could also be a positive effect of getting more “correct” answers during the training sessions, on the performance level. If the panelist gets more “correct” answers, meaning a higher frequency of hitting the target range, then the panelist is potentially more motivated to achieve an even higher frequency of correct responses. This could be the reason why the self-calibrated panel had a constant lower distance from target. 

Even though the self-calibrated panel performed best, the improvement in performance over the training sessions was similar for both panels, indicating that they experienced a similar learning effect over time. It is therefore possible that the performance difference between panels could be due to other reasons. For example, the fixed panel was trained in the morning, a time during the day in which beer is normally not consumed, whereas the self-calibrated panel was trained in the afternoon, a more appropriate time to consume beer. This difference in time of day could have influenced the performance of the panels. However, this is only the case for the training sessions, while there was no difference in time of day for the final DA. The self-calibrated panel also performed the best during the final DA, indicating that the difference in performance between panels is because the self-calibrated panel had a higher starting performance level.

Looking at the accuracy performance over training sessions (distance from target) for the fixed panel only, a small increase in distance from target from TDA 3 to TDA 4, and a decrease again for TDA 5 was observed. This small increase in distance from the target could indicate what Herzog and Fahle [30] define as an oscillating effect. Even though the results of Herzog and Fahle [30] showed that feedback “smoothened” the oscillating effect over replication sessions, they still observed very small increases and decreases in performance for the two feedback conditions with the highest learning effect (trial-by-trail and block type).

The ANOVA indicated an attribute/session interaction effect, and it is clear that some attributes decreased in distance from the target over the training sessions, while others did not. Initially, no change in distance from target could be interpreted as a lack of increase in performance. However, upon further inspection, these attributes were initially already scored very close to the target score, leaving almost no room for improvement. The scores in Table 3 for these three attributes (fruity flavor, sweet taste, and alcoholic aftertaste) indeed show a very small to no decrease in distance from target from training TDA 2 to TDA 6, indicating a lack of learning effect for these attributes. However, all these attributes showed starting distance values (scores on a 100-point line scale for session TDA 2) close to the final performance, 16 for fixed and 10 for self-calibrated, respectively (Figure 4). This indicates very high accuracy for these attributes, making it more difficult to even further enhance the performance.

### 4.3. Comparison of Optimal Target scores and Panel Means Progression over Training Sessions

The target values that these analyses are based upon are not similar between the two panels, as the self-calibrated panel generated their own target values, which changed over training sessions. If one assumes that there is in fact such a thing as an optimal target value, then the nature of the self-calibrated target values could cause an issue. The target values for the self-calibrated panel are based on the mean values from the previous evaluation session, allowing the panel to move away from the “optimal” target scores (which are, in this case, the same as the target scores for the fixed panel). As for the self-calibrated panel, the “true” values are unknown; the panel participants received feedback only based on their own scores, and therefore converged onto their “incorrect” target scores. This potential issue is important to investigate since the effectiveness of FCM is dependent on the truth of the target values being used. This was noted by the developers of the method [39,40], who also stated that incorrect target values (i.e., values not reflecting the real underlying sensory characteristics) cause FCM to be inefficient and could lead to confusion among panelists.

Overall, the fixed panel hit the optimal target values more often and had more attributes where the mean value was moving toward the target, as opposed to converging or moving away compared to the self-calibrated panel (Figure 5). It is clear to see that there are in fact attributes for which the self-calibrated panel’s mean score was moving away from the optimal target instead of toward (e.g., Figure 6b flavor intensity and body mouthfeel). An example of this is the flavor intensity scores for the Jacobsen beer sample. None of the panels were hitting the target value, but the fixed panel could potentially reach the target with more training sessions, as they are moving in the right direction. On the contrary, the self-calibrated panel obtained a target value for flavor intensity, which was much lower than the optimal target value, as their target remained low, and would therefore probably never have hit the optimal value. The same issue is the case for the bitter-taste intensity for the Jacobsen beer, where the self-calibrated panel’s target value moved away from the optimal target value.

The authors are aware that the opposite situation was also observed, where the fixed panel moved away from the optimal target score (Figure 6b sulfury flavor); however, this should, in theory, be fixed if the panel received more training, as the target score was not changing and the panel therefore should have moved toward the optimal target eventually. It would be interesting to investigate this target progression situation over a longer span of a training session, to test this hypothesis. 

If the self-calibrated target ranges end up being different from the optimal target scores, and the optimal target scores are not always known, then the next question is how to define the optimal target scores for unknown products.

The panels used in this study were both relatively new to performing sensory descriptive analysis, and new to evaluating beer. Studies have shown that product-specific knowledge is important for panel performance when evaluating beer [9]. Figure 5 shows that the fixed panels’ mean score lines for each attribute are more diverse across training sessions, compared to the self-calibrated panel’s more “flat” lines (Figure 5a1 vs. Figure 5a2), indicating that the self-calibrated panel had difficulties differentiating between the attributes. It is therefore possible that the issue with the self-calibrated panel’s moving away from the target scores could be solved with a more trained panel. It would therefore be interesting to investigate whether a well-trained and a newly trained sensory panel, both trained using self-calibrated ranges, generate the same target values for the same products. This could shed more light on the possible issues with using self-calibrated target ranges and indicate how the panel’s level of training influences the progression of the target value over training sessions. 

In situations with known products, where fixed scoring targets are possible, this training option should be used. This is the case when industrial panels perform quality control tests, or when new panelists are added to an already existing panel. Moreover, if a company wants to expand its sensory analysis to a new location and thereby recruit new panelists in this location, the new panel could be trained on the fixed scoring ranges of the existing panel, to align both panels in their assessments. 

Based on the GPA results, one could argue that the choice of target value calibration method does not matter. However, if the authors’ theory regarding the progression of target values for the self-calibrated panel is correct, then the sensory profiles generated by the two panels over time will become more and more different, and the choice of target value calibration method will therefore become more important.

### 4.4. Application of FCM and Perceptual Learning Theory

When applying FCM, the panelists receive immediate feedback on the scoring of the sample under evaluation. This type of feedback is defined by Herzog and Fahle [30] as trial-by-trail feedback. These authors found that this type of feedback increases the perceptual learning significantly over replication session. This is in agreement with the results of Findlay et al. [39,40], as well as the results of this current study, where the performance of both panels increased significantly over the training session due to the use of FCM (Figure 4). Additionally, Herzog and Fahle [30] found that a block type of feedback (percentage of correct answers) increased the performance in a similar manner as the trial-by-trial feedback. It is therefore questioned whether a block type of FCM feedback (shown as the overall percentage of hits per product, replication session, or attribute) could increase the performance in a similar manner to a trial-by-trial type of FCM. The task in the study by Herzog and Fahle [30] included only the direction of the offset (left/right), and therefore the outcome could only be right or wrong, and consequently, it did not account for the magnitude of the offset. The feedback given in FCM provides panelists with a “right–wrong” type of answer (inside/outside of target range), as well as information about the distance from the target. Trowbridge and Cason [33] showed that feedback in the type of “right–wrong” was less effective compared to feedback about the distance from the target. It would therefore be interesting to investigate if a block type of feedback by FCM (not including the distance information) yields the same increase in performance compared to the traditional type of feedback, including both right–wrong and distance information.

## 5. Conclusions

Overall, the choice of target value calibration method did not influence the positioning of the samples in the sensory space, as both sensory panels generated very similar sensory profiles. Furthermore, the investigation of the panels’ repeatability and discriminability showed that the self-calibrated panel had the highest repeatability, but that the discriminability was similar. 

The results of average distance from target and standard deviation showed that the self-calibrated panel had the lowest distance from target and standard deviation throughout all sessions, indicating a better accuracy and precision, and thereby a better performance. However, the decrease in average distance from target and standard deviations over training sessions was similar among panels, meaning that the increase in performance was similar, but that the fixed panel had an overall lower performance level, i.e., accuracy and precision, from the beginning of training onward. Furthermore, the use of self-calibrated ranges could be introducing an issue with the progression of the target scores over session, which is why the fixed target ranges seem to be the safest choice, if available. To conclude, the effect of applying fixed versus self-calibrated scoring targets and ranges should be investigated further to fully understand the implications of applying one over the other, as results showed a similar increase in performance and similar sensory configurations between the two panels, while, at the same time, they indicated a potential issue with using self-calibrated ranges. We therefore suggest that researchers use fixed ranges, when they are available.

## Figures and Tables

**Table 1:** (**a**) Samples used in the study for training (T), training descriptive analysis sessions during the training period (TDA), and DA evaluation sessions (E). ABV = ethanol content (% *v*/*v*). (**b**) List of sensory attributes and reference beers used to establish minimum and maximum attribute intensities. Minimum intensity refers to 0–25% of the scale, while maximum intensity refers to the 75–100%.

**(a)**	**Product name**	**Sample code**	**Beer type**	**ABV**	**Session**
	Tuborg Gold	Gold	Strong Pilsner	5.6	T
	Budweiser	Budweiser	Strong Pilsner	5.0	T
	Carlsberg Elephant	Elephant	Strong Pilsner	7.2	T
	Carlsberg Nordic Golden	Nordic	Pilsner, Alcohol free	0.5	T
	Tuborg Green	Tuborg	Pilsner	4.6	T, TDA, and E
	Carlsberg Pilsner	Carlsberg	Pilsner	4.6	T, TDA, and E
	Jacobsen Extra Pilsner	Jacobsen	Pilsner, Special brew	5.5	T, TDA, and E
	Tuborg Super Light	Tuborg SL	Pilsner, Alcohol free	0.09	T, TDA, and E
	Heineken	Heineken	Pilsner	4.6	T, TDA, and E
**(b)**	**Modality**	**Attribute**	**Minimum Intensity** **(0–25 on 100-point scale)**	**Maximum Intensity** **(75–100 on 100-point scale)**	
	Flavor (F)	Intensity	Tuborg SL	Jacobsen	
	Flavor (F)	Fruity	Carlsberg	Gold	
	Flavor (F)	Malty	Budweiser	Elephant	
	Flavor (F)	Hoppy	Tuborg SL	Jacobsen	
	Flavor (F)	Sulfury	Tuborg SL	Elephant	
	Basic Taste (T)	Sweetness	Carlsberg	Nordic	
	Basic Taste (T)	Sourness	Nordic	Tuborg SL	
	Basic Taste (T)	Bitterness	Budweiser	Jacobsen	
	Mouthfeel (Mf)	Body	Tuborg SL	Elephant	
	Aftertaste (Af)	Alcoholic	Nordic	Elephant	
	Aftertaste (Af)	Lingering	Tuborg SL	Jacobsen	

**Table 2:** (**a**) Overview of activities performed during the different nine training sessions, T1 to T9. (**b**) Overview of samples included during the training descriptive analysis and application of FCM during these sessions.

**(a)**	**Session**		**Description of activity**					
	T1	1.	Presentation of basic taste solutions in DI water ^1^: sweet, sour, bitter, and salty.					
		2.	Presentation of spiked beer samples. ^2^					
		3.	Comparison between basic taste solutions and spiked samples.					
	T2	1.	Presentation of spiked beer samples.					
		2.	Presentation of beer reference frame for attribute minima and maxima. ^3^					
		3.	Comparison between spiked samples and reference samples.					
	T3	1.	Repetition of beer reference frame and reference samples.					
		2.	Booth evaluation: TDA ^4^ 1.					
	T4	1.	Individual evaluation of three beer samples, Jacobsen (1 rep) and Tuborg SL (2 rep), on paper ballots.					
		2.	Discussion of results in reference to placing the samples on four sections of the scale: low = 0–25%, medium-low = 25–50%, medium-high = 50–75%, high = 75–100%.					
	T5	1.	Repetition of beer reference frame and reference samples.					
		2.	Booth evaluation: TDA 2.					
	T6	1.	Individual generation of word associations for the different attributes on paper ballots.					
		2.	Discussion of word associations in plenum.					
		3.	Booth evaluation: TDA 3.					
	T7	1.	Repetition of word associations.					
		2.	Repetition of beer reference frame and reference samples.					
		3.	Comparison between reference samples and spiked samples (instructed to exclude intensity).					
		4.	Booth evaluation: TDA 4.					
	T8	1.	Presentation and discussion of results from TDA 4.					
		2.	In booth: quiz with identification of reference samples. Low and high attribute intensity sample presented for each attribute, and panelists had to indicate which sample was respectively high and low in the particular attribute.					
		3.	Booth evaluation: TDA 5.					
	T9	1.	Repetition of beer reference frame and reference samples.					
		2.	Booth evaluation: TDA 6.					
**(b)**	**Product name**		**TDA 1**	**TDA 2**	**TDA 3**	**TDA 4**	**TDA 5**	**TDA 6**
	Tuborg		1 rep ^5^	1 rep *	2 rep *		1 rep	2 rep *
	Carlsberg			1 rep	2 rep *		2 rep *	2 rep *
	Jacobsen			1 rep			1 rep *	1 rep *
	Tuborg SL		1 rep	1 rep *		2 rep *	1 rep *	
	Heineken		1 rep	1 rep		1 rep *		1 rep

^1^ Sweet: 16 g/L sucrose (Domino Foods, Yonkers, NY); sour: 1.5 g/L citric acid (Sigma-Aldrich, St. Louise, MO); bitter: 0.034 µL/mL Iso-α-acids (Isohop^®^, Barth-Haas Group, Nürnberg, Germany); salt: 3 g/L sodium chloride (Sigma-Aldrich, St. Louise, MO). ^2^ Bitter: 0.023 µL/mL Iso-α-acids (Isohop^®^, Barth-Haas Group, Nürnberg, Germany) dissolved in Heineken. Malt: Isobutyraldehyde, 1 aroxa^TM^ flavor capsule dissolved in 990 mL Heineken. Hoppy: hop oil extract, 1 aroxa^TM^ flavor capsule dissolved in 990 mL Heineken. Fruity: isoamyl acetate, 1 aroxa^TM^ flavor capsule dissolved in 990 mL Heineken. Sulfur: hydrogen sulfide, 1 aroxa^TM^ flavor capsule dissolved in 990 mL Heineken. ^3^ See Table 1 for beer reference frame. ^4^ TDA = training descriptive analysis. The numbers refer to the TDA sessions 1–6. ^5^ Notions “1 rep” and “2 rep” indicate samples that were presented once or twice in a single training session. * Indicates that FCM was applied for all attributes for this sample, but only for one of the two duplicates.

**Table 3:** Average distance from target per attribute and panel. The + indicates a performance improvement, the ÷ indicates a performance decrease, and the = indicates an unaltered performance, compared to the prior TDA session. An unaltered performance is a difference lower than 0.5 on a 100-point scale.

		Fixed					Self-Calibrated				
		TDA 2	TDA 3	TDA 4	TDA 5	TDA 6	TDA 2	TDA 3	TDA 4	TDA 5	TDA 6
**Flavor**	**Intensity**	38.6	14.4	20.1	15.0	13.2	22.7	14.6	12.9	9.0	8.5
			+	÷	+	+		+	+	+	+
	**Fruit**	14.5	21.2	15.5	11.8	22.2	8.9	12.7	8.7	11	8.6
			÷	+	+	÷		÷	+	÷	+
	**Hoppy**	28.3	18.9	21.3	12.9	14.6	27.3	15.8	14.6	13.0	9.8
			+	÷	+	÷		+	+	+	+
	**Malt**	26.7	20.3	12.3	14.3	14.0	26.2	13.8	15.2	11.5	9.0
			+	+	÷	=		+	÷	+	+
	**Sulfury**	13.1	16.9	13.5	15.7	12.8	14.4	11.5	14.5	6.5	5.7
			÷	+	÷	+		+	÷	+	+
**Basic Taste**	**Sweet**	14.4	16.4	14.2	14.4	18.6	11.8	20.5	9.7	8.8	9.2
			÷	+	=	÷		÷	+	+	=
	**Sour**	36.8	19.3	27.3	23.5	17.4	19.1	17.8	14.5	12.6	9.4
			+	÷	+	+		+	+	+	+
	**Bitter**	23.7	22.1	13.8	23.0	19.5	19.2	16.4	17.1	19.2	13.8
			+	+	÷	+		+	÷	÷	+
**Mouthfeel**	**Body**	24.1	16.3	23.9	20.8	13.9	17.2	14.8	14.1	10.7	12.1
			+	÷	+	+		+	+	+	+
**Aftertaste**	**Alcoholic**	14.3	18.0	24.5	20.6	13.4	9.7	13.8	13.4	9.7	9.4
			÷	÷	+	+		÷	=	+	=
	**Lingering**	16.8	19.0	28.3	17.5	15.6	18.1	13.4	14.0	11.9	10.7
			÷	÷	+	+		+	÷	+	+

## References

[1] Lawless H.T., Heymann H. (2010). Sensory Evaluation of Food: Principles and Practices.

[2] Meilgaard M.C., Civille G.V., Carr B.T. (1999). Sensory Evaluation Techniques.

[3] Dijksterhuis G.B., Byrne D.V. (2005). Does the mind reflect the mouth? Sensory profiling and the future. Crit. Rev. Food Sci. Nutr..

[4] Murray J.M., Delahunty C.M., Baxter I.A. (2001). Descriptive sensory analysis: Past, present and future. Food Res. Int..

[5] Cairncross S.E., Sjöström L.B. (1950). Flavor Profiles: A new approach to flavor problems. Food Technol..

[6] Brandt M.A., Skinner E.Z., Coleman J.A. (1963). Texture Profile Method. J. Food Sci..

[7] Stone H., Sidel J.L., Oliver S., Woolsey A., Singleton R.C. (1974). Sensory analysis by quantitative descriptive analysis. Food Technol..

[8] Stone H., Sidel J.L. (1993). Sensory Evaluation Practices.

[9] Elgaard L., Jensen S., Mielby L.A., Byrne D.V. (2019). Performance of beer sensory panels: A comparison of experience level, product knowledge and responsiveness to feedback calibration. J. Sens. Stud..

[10] Elgaard L., Mielby L.A., Heymann H., Byrne D.V. (2019). Effect of product involvement on panels’ vocabulary generation, attribute identification and sample configurations in beer. Foods.

[11] Bitnes J., Rødbotten M., Lea P., Ueland Ø., Martens M. (2007). Effect of product knowledge on profiling performance comparing various sensory laboratories. J. Sens. Stud..

[12] Chambers D.H., Allison A.-M.A., Chambers IV E. (2004). Training effects on performance of descriptive panelists. J. Sens. Stud..

[13] Giacalone D., Ribeiro L., Frøst M. (2016). Perception and Description of Premium Beers by Panels with Different Degrees of Product Expertise. Beverages.

[14] Chollet S., Valentin D., Abdi H. (2005). Do trained assessors generalize their knowledge to new stimuli?. Food Qual. Prefer..

[15] Zamora M.C., Guirao M. (2004). Performance comparison between trained assessors and wine experts using specific sensory attributes. J. Sens. Stud..

[16] Byrne D.V., Bak L.S., Bredie W.L.P., Bertelsen G., Martens M. (1999). Development of a sensory vocabulary for warmed-over flavor: Part I. In porcine meat. J. Sens. Stud..

[17] Drake M.A., Civille G.V. (2002). Flavor Lexicons. Compr. Rev. Food Sci. Food Saf..

[18] Lawless L.J.R., Civille G.V. (2013). Developing lexicons: A review. J. Sens. Stud..

[19] Byrne D.V., Bredie W.L.P., Martens M. (1999). Development of a sensory vocabulary for warmed-over flavor: Part II. In chicken meat. J. Sens. Stud..

[20] Suwonsichon S. (2019). The importance of sensory lexicons for research and development of food products. Foods.

[21] Yang J., Lee J. (2019). Application of sensory descriptive analysis and consumer studies to investigate traditional and authentic foods: A review. Foods.

[22] Tran T., James M.N., Chambers D., Koppel K., Chambers IV E. (2019). Lexicon development for the sensory description of rye bread. J. Sens. Stud..

[23] Chambers IV E., Sanchez K., Phan U.X.T., Miller R., Civille G.V., Di Donfrancesco B. (2016). Development of a “living” lexicon for descriptive sensory analysis of brewed coffee. J. Sens. Stud..

[24] Dijksterhuis G. (1995). Assessing Panel Consonance. Food Qual. Prefer..

[25] Martens M., Bredie W.L., Martens H. (2000). Sensory profiling data studied by partial least squares regression. Food Qual. Prefer..

[26] Qannari E.M., MacFie H.J.H., Courcoux P. (1999). Performance indices and isotropic scaling factors in sensory profiling. Food Qual. Prefer..

[27] Tomic O., Nilsen A., Martens M., Næs T. (2007). Visualization of sensory profiling data for performance monitoring. LWT Food Sci. Technol..

[28] Goldstone R.L. (1998). Perceptual learning. Annu. Rev. Psychol..

[29] Bangert-Drowns R.L., Kulik C.-L.C., Kulik J.A., Morgan M. (1991). The Instructional Effect of Feedback in Test-Like Events. Rev. Educ. Res..

[30] Herzog M.H., Fahle M. (1997). The role of feedback in learning a vernier discrimination task. Vis. Res..

[31] Kulik J.A., Kulik C.-L.C. (1988). Timing of Feedback and Verbal Learning. Rev. Educ. Res..

[32] Shute V.J. (2008). Focus on Formative Feedback. Rev. Educ. Res..

[33] Trowbridge M.H., Cason H. (1932). An experimental study of thorndike’s theory of learning. J. Gen. Psychol..

[34] Walk R.D. (1966). Perceptual learning and the discrimination of wines. Psychon. Sci..

[35] Spence C. (2019). Perceptual learning in the chemical senses: A review. Food Res. Int..

[36] Lestringant P., Delarue J., Heymann H. (2019). 2010–2015: How have conventional descriptive analysis methods really been used? A systematic review of publications. Food Qual. Prefer..

[37] Kluger A.N., DeNisi A. (1996). The effects of feedback interventions on performance: A historical review, a meta-analysis, and a preliminary feedback intervention theory. Psychol. Bull..

[38] Kulhavy R.W., Wager W., Dempsey J.V., Sales G.C. (1993). Feedback in programmed instruction: Historical context and implications for practice. Interactive Instruction and Feedback.

[39] Findlay C.J., Castura J.C., Schlich P., Lesschaeve I. (2006). Use of feedback calibration to reduce the training time for wine panels. Food Qual. Prefer..

[40] Findlay C.J., Castura J.C., Lesschaeve I. (2007). Feedback calibration: A training method for descriptive panels. Food Qual. Prefer..

[41] Richards M., De Kock H.L., Buys E.M. (2014). Multivariate accelerated shelf-life test of low fat UHT milk. Int. Dairy J..

[42] Acevedo W., Capitaine C., Rodríguez R., Araya-Durán I., González-Nilo F., Pérez-Correa J.R., Agosin E. (2018). Selecting optimal mixtures of natural sweeteners for carbonated soft drinks through multi-objective decision modeling and sensory validation. J. Sens. Stud..

[43] Espinoza M.I., Vincken J.P., Sanders M., Castro C., Stieger M., Agosin E. (2014). Identification, quantification, and sensory characterization of teviol glycosides from differently processed stevia rebaudiana commercial extracts. J. Agric. Food Chem..

[44] Chang A.C., Dando R. (2018). Exposure to light-emitting diodes may be more damaging to the sensory properties of fat-free milk than exposure to fluorescent light. J. Dairy Sci..

[45] Martin N., Carey N., Murphy S., Kent D., Bang J., Stubbs T., Wiedmann M., Dando R. (2016). Exposure of fluid milk to LED light negatively affects consumer perception and alters underlying sensory properties. J. Dairy Sci..

[46] Fisher C.M., King S.K., Castura J.C., Findlay C.J. (2016). Does data collection device affect sensory descriptive analysis results?. J. Sens. Stud..

[47] Obst K., Paetz S., Backes M., Reichelt K.V., Ley J.P., Engel K.-H. (2013). Evaluation of unsaturated alkanoic acid amides as maskers of epigallocatechin gallate astringency. J. Agric. Food Chem..

[48] Bavay C., Symoneaux R., Maître I., Kuznetsova A., Brockhoff P.B., Mehinagic E. (2013). Importance of fruit variability in the assessment of apple quality by sensory evaluation. Postharvest Biol. Technol..

[49] Pietrasik Z., Wang H., Janz J.A.M. (2013). Effect of canola oil emulsion injection on processing characteristics and consumer acceptability of three muscles from mature beef. Meat Sci..

[50] Mahan E.D., Morrow K.M., Hayes J.E. (2011). Quantitative perceptual differences among over-the-counter vaginal products using a standardized methodology: Implications for microbicide development. Contraception.

[51] Williams E.J. (1949). Experimental designs balanced for the estimation of residual effects of treatments. Aust. J. Sci. Res..

[52] Gower J.C. (1975). Generalized procrustes analysis. Psychometrika.

[53] Le S., Josse J., Husson F. (2008). FactoMineR: An R package for multivariate analysis. J. Stat. Softw..

[54] Xiong R., Blot K., Meullenet J.F., Dessirier J.M. (2008). Permutation tests for generalized procrustes analysis. Food Qual. Prefer..

[55] Dijksterhuis G. (1995). Multivariate data analysis in sensory and consumer science: An overview of developments. Trends Food Sci. Technol..

[56] Hervé M. RVAideMemoire: Testing and Plotting Procedures for Biostatistics. https://CRAN.R-project.org/package=RVAideMemoire.

[57] Castura J.C., Findlay C.J., Lesschaeve I. (2005). Monitoring calibration of descriptive sensory panels using distance from target measurements. Food Qual. Prefer..

[58] Lenth R., Singmann H., Love J., Buerkner P., Hervé M. emmeans: Estimated Marginal Means, aka Least-Squares Means. https://CRAN.R-project.org/package=emmeans.

[59] Piepho H.P. (2004). An algorithm for a letter-based representation of all-pairwise comparisons. J. Comput. Graph. Stat..

[60] Mangiafico S. rcompanion: Functions to Support Extension Education Program Evaluation. https://CRAN.R-project.org/package=rcompanion.

[61] Wickham H. (2016). ggplot2: Elegant Graphics for Data Analysis.

